# Napping during the night shift and recovery after work among hospital nurses^1^

**DOI:** 10.1590/0104-1169.0147.2532

**Published:** 2015

**Authors:** Thaís Aparecida de Castro Palermo, Lúcia Rotenberg, Regina Célia Gollner Zeitoune, Aline Silva-Costa, Ester Paiva Souto, Rosane Härter Griep

**Affiliations:** 2 Master´s student, Escola de Enfermagem Anna Nery, Universidade Federal do Rio de Janeiro, Rio de Janeiro, RJ, Brazil; 3 PhD, Researcher, Instituto Oswaldo Cruz, Rio de Janeiro, RJ, Brazil; 4 PhD, Full Professor, Escola de Enfermagem Anna Nery, Universidade Federal do Rio de Janeiro, Rio de Janeiro, RJ, Brazil; 5 Doctoral student, Escola Nacional de Saúde Pública, Fundação Oswaldo Cruz, Rio de Janeiro, RJ, Brazil

**Keywords:** Nurses, Male, Nurses, Night Work, Sleep, Occupational Health

## Abstract

**OBJECTIVE:**

To analyze the association between the length of napping during the night shift and the recovery after work among nurses.

**METHOD:**

Cross-sectional epidemiological study involving 1940 nurses from 18 public hospitals in the City of Rio de Janeiro. A multidimensional and self-applied questionnaire was used with information about health, sociodemographic and occupational characteristics, health-related behaviors and housework. Multiple logistic regression was applied to identify the association, adjusted for confounding variables.

**RESULTS:**

The gross analyses showed 44%, 127% and 66% higher chances of a high level of recovery after work for nurses who sleep up to two hours, between 2.1 and 3 hours and 3.1 hours or more, respectively, when compared to the nurses who do not sleep. After adjusting for confounding variables, the association only continues significant for the group that sleeps 2.1 to 3 hours during the night shift (OR=1.79; 95%CI=1.33-2.41).

**CONCLUSION:**

The association between the length of napping and the high level of recovery after work, confirmed in the present results, can be included in the studies that aim to support more appropriate policies aimed at improving the workers' work, life and health conditions, not only in nursing, but night-shift workers in general.

## Introduction

Work produces effort that reflects in emotional, cognitive and behavioral symptoms, which take some time to revert^(^ 1 ^)^. With appropriate time and sufficient possibilities of recovery (during and after work), the return to work will happen without the residual symptoms of previous efforts. When the recovery is insufficient and cumulative, however, the need for recovery increases and is gradually replaced by more severe symptoms, related to long-term fatigue and illness^(^ 2 ^)^. The need for recovery after work is characterized as an intermediary position between the demands of work and the future health condition and is related to the initial phase of the continuing process that can lead to chronic fatigue^(^ 3 ^)^.

Hospitals function uninterruptedly, which implies work schemes in day and night shifts. An essential health aspect that needs to be analyzed with regard to shift workers, especially in the night shift, refers to the influence of the work hours on the quality and length of sleep^(^ 4 ^)^ and, in turn, on the recovery after work^(^ 5 ^)^. Sleep, in terms of quality and length, varies at different times during 24 hours. Daytime sleep, usually adopted by nighttime workers, tends to be worse and shorter^(^ 6 ^)^ when compared to nighttime sleep.

In that perspective, the reduced length and sleep quality changes in nursing professionals working night shifts have been demonstrated in Brazilian^(^ 6 ^-^ 7 ^)^ and international studies^(^ 4 ^)^. The reduced length of sleep and, more severely, the deprival from sleep entail greater risks of injuries and occupational accidents, as well as harm for these workers' health and quality of life^(^ 7 ^)^, in function of the greater sleepiness at work and reduced state of alertness deriving from nighttime work^(^ 6 ^)^.

One way to cope with this partial deprival from sleep is to nap during nighttime work^(^ 8 ^)^. Defined as any sleep period shorter than 50% of the average length of nighttime sleep^(^ 9 ^)^, napping during the night shift is associated with lower levels of sleepiness during work^(^ 8 ^)^, maintenance of alertness and performance^(^ 10 ^)^ and can compensate for the short length of sleep at home among nighttime workers^(^ 11 ^)^.

In a context of nursing workers, predominantly female, nighttime naps can be even more relevant for workers with a housework overload, which implies less time for daytime sleep during the days off^(^ 12 ^)^. In addition, the 12-hour-work schemes followed by 36 or 60 hours of rest^(^ 12 ^-^ 13 ^)^, which allow these professionals to be active in more than one productive activity, as well as the long workdays of the Brazilian nursing professionals, can interfere in the time available for sleep^(^ 12 ^)^.

The importance of napping during the night shift for the recovery after work has been demonstrated in a study in the context of Brazilian nursing^(13), ^which identified the association between napping and better recovery, provided that the work journey at home does not exceed ten hours per week. This study intends to complement research on the importance of napping during nighttime work for the recovery after work in an expanded sample and considering a heterogeneous population in terms of sex.

The objective in this article was to analyze the association between the length of napping during the night shift and the recovery after work among nurses working at the main public hospitals in Rio de Janeiro.

## Method

### Description of study design and sample

This article uses data from the Nurses' Health Study. A cross-sectional epidemiological study was undertaken in the 18 largest public hospitals in the city of Rio de Janeiro, RJ. The data were collected between 2010 and 2011, through the application of a structured, multidimensional and self-applied questionnaire. The 18 largest public hospitals in the city of Rio de Janeiro, under federal, state and municipal management, took part in the study. These hospitals were visited, scheduling meetings with the nursing heads to disseminate the study. The list of the name and work sector was requested for the purpose of a face-to-face approach. The nurses received the questionnaires, together with an invitation letter to participate and the Informed Consent Form (ICF). After signing the ICF, the nurses completed and sealed the questionnaire and handed it over to one of the study collaborators. The data analyses were done in SPSS (v.19). In this paper, data were considered from 1940 nurses (61.4% of all nurses contacted) who worked during night shifts.

### Exposure variable: length of napping during night shift

This variable was assessed through the following question: "As regards the time to sleep or rest during the shift, would you say that, in most cases: "you only rest"; "you sleep for about ___h___min"; "you neither sleep nor rest". The variable was categorized in four levels: (i)"does not sleep" (reference category), (ii)"sleeps up to 2 hours", (iii)"sleeps between 2.1 and 3 hours" and (iv)"sleeps 3.1 hours or more". The groups that reported that they "neither sleep nor rest" and "only rest" were included under the category of the nurses who do not sleep.

### Outcome variable: recovery after work

The recovery after work was assessed using the short version of the scale of Need for Recovery - NFR. This instrument assesses short-term effects of work induced by fatigue, such as lack of attention, irritability, social isolation, reduced performance and quality of recovery time after work^(^ 1 ^,^ 3 ^)^. The original scale, which consists of eleven items, was adapted to Brazilian Portuguese^(^ 14 ^)^. In this study, the short version of the scale was used, recently proposed by the author of the scale and forwarded through personal communication. This version consists of six items from the original version with Likert-style answers (1-4), with scores ranging between "always" and "never", which include fatigue-related aspects after a workday. The items were grouped in a score that was obtained by adding up the scores obtained on the set of scale items, which ranged between 6 and 24. The score was categorized in recovery tertiles, with scores 6-13 being considered as low recovery; scores 14-17 medium recovery and scores 18-24 high recovery. After this categorization, the medium and high categories were grouped, resulting in only two categories: low (reference category) and high recovery (grouping the medium and high scores).

### Co-variables

Sociodemographic, occupational and health behavior-related variables were included: gender, age (grouped in "23 to 35 years", "36 to 45 years" and "46 to 67 years"), marital situation (grouped in "married", "separated, divorced or widowed" and "single"), *per capita *family income ("up to R$ 1,394.83", "from R$ 1,400.90 till R$ 2,324.50" and "from R$ 2,324.83 till R$ 7,440.00"), presence of children under six years of age), weekly hour load of professional work (grouped in "up to 30 hours", "from 31 till 60 hours", "more than 61 hours"), domestic overload (grouped in "low" and "high" overload), length of nighttime work in nursing (categorized in "less than 1 year", "1 to 5 years", "6 to 10 years", "11 to 20 years" and "21 years or more"), permission to sleep during the night shift, napping time, alcohol consumption (grouped in "does not drink", "low risk", "medium risk" and "high risk"), complaints of insomnia and physical exercises.

### Statistical analyses

The psychometric performance of the short version of the scale of need for recovery after work was assessed, as this is the first time the instrument was used in the Brazilian context. The dimensional structure and internal consistency were assessed with the help of exploratory factor analysis, using the software Mplus - version 7.1, adopting Geomin rotation and the robust weighted least squares (WLSMV) estimator, appropriate for categorical or ordinal variables (items).

To assess the appropriateness of the model, three adjustment rates were used; the comparative fit index (CFI), the Tucker-Lewis index (TLI) and the root mean square error of approximation (RMSEA). The comparative fit index and the Tucker-Lewis index assess the incremental adjustment of the model in comparison with a null model. Both range between 0 and 1, and values superior to 0.90 indicate appropriate adjustment^(^ 15 ^)^.

The root mean square error of approximation (RMSEA) is an adjustment index that assesses the parsimony of the model proposed in relation to the number of estimated coefficients. It is a measure that tries to correct the trend of the chi-square statistics to reject any model specified with a sufficiently large sample. Coefficients superior to 0.06 can be considered appropriate^(^ 15 ^)^.

The internal consistency, which indicates whether the items represent a shared theoretical construct, is frequently verified using Cronbach's alpha coefficient as a reliability measure^(^ 15 ^)^. In this study, however, the decision was made to use the compound reliability, a reliability coefficient based on structural equations modeling, with a view to overcoming the limitations of Cronbach's alpha, which underestimates the reliability of the scale, like in situations in which the premise of *tau-*equivalence is violated for example^(^ 15 ^)^. The compound reliability assesses whether the items present internal consistency and, for that measure, coefficients of 0.7 or higher are considered satisfactory. It is based on the estimated factor loadings, variance and covariance of measuring errors.

Univariate and bivariate analyses were applied to verify the association between the exposure variable and the outcome variable. For the multiple analyses, in turn, multiple logistic regression was used, in the attempt to identify the association adjusted by confounding variables.

### Ethical aspects

In compliance with the procedures recommended in Resolution 196/96, the study was submitted to and received approval from the Research Ethics Committee - CEP Fiocruz under number 472/08 and from the participating hospitals. Authorization was obtained form the nursing service head at each hospital. The participants were asked to sign the Informed Consent Form (ICF) for their participation and for the publication of the results, guaranteeing the subjects' anonymity. These analyses were submitted to the Research Ethics Committee at Anna Nery School of Nursing/UFRJ and received approval under number 191.240.

## Results

Among the nurses assessed, female nurses were predominant (84.7%). In total, 44.6% were 35 years or older (mean age 38 years, SD=9.4 years and range from 23 till 67 years). The majority was married, with a mean *per capita* income of R$ 2,101.41 (SD=R$ 1,454.21) and 19.1% indicated having children under six years of age. The mean weekly hour load of professional work was 61 hours per week (SD=21.7 hours), ranging from 5 to 132 hours per week; 46.5% mentioned a weekly hour load of professional work of 61 hours or more. About 29.5% of the nurses showed a high domestic burden.

As regards the length of nighttime work in Nursing, the mean length was 9 years (SD=7.8 years), with 44.4% having worked less than 5 years in the night shift. Almost all participants (97.6%) mentioned having permission to sleep during the night shift. Almost half indicated not sleeping though (46.6%), 26.9% slept between 2.1 and 3 hours (121 to 180 minutes) and 19.4% slept up to two hours. The mean length of the naps during the night shift was 159 minutes, with a standard deviation of 47.5 minutes and range from 60 till 390 minutes.

About 30% of the participants showed a medium/high risk in terms of alcohol consumption, one third reported complaints of insomnia and almost 70% indicated no physical exercise during the two weeks before the data collection, in accordance with Table 1.

**Table 1 -:** Characterization of the nurses who participated in the Study of Nurses' Health (n=1940). Rio de Janeiro, RJ, Brazil, 2010-2011

Variables		n	%
Sex			
	Female	1644	84.7
	Male	296	15.3
Age			
	From 23 till 35 years	853	44.6
	From 36 till 45 years	642	33.5
	From 46 till 67 years	419	21.9
Marital Situation			
	Married	1091	56.9
	Separated, Divorced and Widowed	343	17.9
	Single	482	25.2
*Per capita *income			
	Up to R$ 1,394.83	561	32.7
	Between R$ 1,400.90 and R$ 2,324.50	614	35.9
	From R$ 2,324.83 till R$ 7,440.00	537	31.4
Presence of children under 6 years of age			
	Yes	362	19.1
	No	1537	80.9
Weekly hour load of professional work			
	Up to 30 hours	160	8.6
	31 till 60 hours	838	44.9
	61 hours or more	869	46.5
Domestic burden			
	Low	1364	70.5
	High	570	29.5
Length of nighttime work in Nursing			
	Less than 1 year	174	9.2
	1 to 5 years	666	35.2
	6 to 10 years	449	23.6
	11 to 20 years	416	22
	21 years or more	189	10
Permission to sleep during the night shift			
	Yes	1869	97.6
	No	45	2.4
During the permitted sleep time			
	Sleeps	994	53.4
	Rests only (unable to sleep)	819	44
	Neither sleeps nor rests	47	2.6
Length of napping			
	Does not sleep	866	46.6
	Sleeps up to 2 hours (120 minutes)	353	19.4
	Sleeps between 2.1 and 3 hours (126 till 180 minutes)	497	26.9
	Sleeps 3.1 hours or more (186 minutes or more)	131	7.1
Alcohol consumption			
	Does not drink	722	38
	Low risk	549	28.8
	Medium risk	547	28.7
	High risk	85	4.5
Complaints of insomnia			
	Yes	639	33.2
	No	1284	66.8
Physical exercise in the past two weeks			
	Yes	584	30.3
	No	1344	69.7

In line with the originally proposed model, the one-dimensional scale showed factor loadings ranging from 0.578 to 0.924. The adjustment ratios were: Comparative fit index (CFI)=0.959, Tucker-Lewis index (TLI)=0.931 and Root mean square error of approximation (RMSEA)=0.223. The compound reliability (CR) equaled 0.90 and the mean extracted variance (MEV) 0.61. The assessment of the modification ratios showed a high correlation between 'b' and 'c' and between 'e' and 'f'. The RMSEA coefficients were, respectively, 0.118 (0.108-0.128) and 0.064 (0.054-0.076) after the specifications of the correlations between 'b' and 'c' and 'e' and 'f", according to Table 2 below.

**Table 2 -:** Confirmatory factor analysis of the short version of the scale of need for recovery after work. Rio de Janeiro, RJ, Brazil, 2010-2011

Variables	Model 1			Model 2			Model 3	
	Loadings	Uniqueness		Loadings	Uniqueness		Loadings	Uniqueness
a- I find it difficult to relax at the end of a workday	0.578	0.666		0.626	0.608		0.634	0.598
b- At the end of the workday I really feel exhausted	0.92	0.153		0.713	0.491		0.736	0.459
c- Because of my work, I feel very tired at the end of the day	0.924	0.146		0.713	0.492		0.735	0.460
d- I find it difficult to pay attention or concentrate during my free time after a workday	0.756	0.429		0.820	0.327		0.847	0.283
e- I find it difficult to take interest in other people as soon as I get home from work	0.763	0.418		0.812	0.341		0.748	0.441
f- When I get home after work I need to be left in peace for some time	0.686	0.53		0.731	0.466		0.653	0.573
Inter-item correlation								
b ßà c				0.770			0.754	
e ßà f				-----			0.363	
Adjustment Indices								
*Comparative fit index* (CFI)	0.959			0.990			0.997	
*Tucker-Lewis index* (TLI)	0.931			0.981			0.994	
*Root mean square error of approximation* (RMSEA)	0.223 (0.213; 0.233)			0.118 (0.108; 0.128)			0.064 (0.054; 0.076)	
Convergent Validity Measures								
Compound reliability (CR)	0.90			0.88			0.92	
Mean extracted variance	0.61			0.55			0.66	

An association was identified between napping during the night shift and the recovery after work. Higher frequencies (82.5%) of high recovery after work were observed among participants who sleep between 2.1 and three hours during the night shift. The group that indicated sleeping more than three hours showed a lower recovery frequency (78.6%) than the group that indicated sleeping between 2.1 and three hours.

Table 3 displays the gross and adjusted analyses of the association between the length of napping during the night shift and the recovery after work. The gross analyses showed ratios 44%, 127% and 66% higher of high recovery after work for professionals who sleep up to 2 hours, between 2.1 and three hours and 3.1 hours or more, respectively, when compared to professionals who do not sleep. After adjusting for sociodemographic variables (Model 2), behaviors and problems related to health and housework (Model 3), the association remains significant for the group that sleeps between 2.1 and three hours during the night shift (OR=1.79; 95%CI=1.33-2.41). In addition, a gradient is observed in the ratios between professionals who sleep up to two hours and between 2.1 and three hours. Nevertheless, no increase is observed in the recovery ratios for professionals who sleep 3.1 hours or more.

**Table 3 -:** Gross and adjusted association between the length of napping during the night shift and the recovery after work for nurses who participated in the Health Study of Nurses (n=1940). Rio de Janeiro, RJ, Brazil, 2010-2011

Length of napping	Model 1	Model 2	Model 3
	Gross OR	Adjusted OR*	Adjusted OR^†^
	(95%CI)	(95%CI)	(95%CI)
Does not sleep	1	1	1
Sleeps up to 2 hours	1.44 (1.07-1.93)	1.42 (1.05-1.90)	1.33 (0.98-1.81)
Sleeps between 2.1 and 3 hours	2.27 (1.71-3.00)	2.20 (1.65-2.93)	1.79 (1.33-2.41)
Sleeps more than 3.1 hours	1.66 (1.06-2.60)	1.91 (1.21-3.02)	1.50 (0.94-2.40)

* Adjusted for sex, age, marital situation and per capita income.

† Additionally adjusted for alcohol consumption, complaints of insomnia, physical exercise and domestic burden.

## Discussion

In this study, napping during the night shift for 2.1 to three hours was associated with greater recovery after work. After adjusting for confounding variables, the association with other classifications of napping time did not continue. Similar results were identified in a study of female nursing professionals, only considering those submitted to short durations of housework^(^ 12 ^-^ 13 ^)^.

The present study results suggest a possible beneficial effect of nighttime napping for two to three hours in terms of recovery after work. The aspects of sleep inertia should be taken into account though, a feeling of lethargy and transitory reduction of cognitive and motor performance that can occur when awaking in function of the previous waking time, the moment of the nap and its length^(^ 8 ^,^ 16 ^)^. In fact, this aspect deserves further analysis, as it can constitute a limiting factor for the implementation of this practice in the context of healthcare.

In this research, naps shorter than two hours seem to be insufficient to guarantee the recovery after work, possibly due to the high workload these workers are submitted to. Our results indicated a mean weekly workload of 61 hours. The extensive work burden in the context of Brazilian nursing workers has been demonstrated^(13,17) ^and is much higher than that observed among nurses from different European countries^(^ 18 ^)^. High burdens can affect both the workers' health and the quality of care delivery^(^ 19 ^)^.

Not only the occurrence of napping influences the recovery after work, but also its length^(^ 13 ^)^. The mean length of napping during the night shift was about two hours and a half (159 minutes), that is, higher than that identified in other studies^(^ 6 ^,^ 11 ^-^ 12 ^)^, which identified means of 120, 138, 141 and 150 minutes, respectively. The high weekly loading among the workers studied here may partially explain the longer length of napping. In view of the possibility of napping during the night shift, the nursing professionals get organized in function of the work demand and the preference of each^(^ 12 ^,^ 20 ^)^. In general, the workers themselves organize the napping times themselves, which are divided in two periods, the first from 00:00h till 03:00h and the second from 03:00h till 06:00h^(^ 6 ^,^ 12 ^,^ 20 ^)^.

Among the workers who mentioned sleeping more than three hours, no higher recovery ratios were observed when compared to those who did not sleep. The great variation in the length of napping observed in the group that slept more than three hours can partially explain these results. In addition, this group displays distinguished characteristics when compared to the other groups. Despite acknowledging that, strictly speaking, this is not about napping during the night shift (the length surpasses the definition of what is considered as a nap), the choice was made to maintain it in the analyses and assess it in the study context, as a considerable number of nurses mentioned this condition.

Although napping during the night shift is not regulated in Brazil, the large majority of the participants had tacit agreements with their respective heads, which allowed them to nap/sleep during the night shift, which was considered common practice in Brazilian public hospitals^(^ 6 ^,^ 11 ^-^ 12 ^,^ 20 ^)^. It should be highlighted that, despite the permission to sleep, 46.6% of the workers indicated that they did not sleep. This finding may be partially related to the fact that many hospitals do not have appropriate sites for nighttime napping, as shown in a qualitative study about nursing workers' perception of the conditions to sleep or rest during the night. The authors showed that the lack of regulation for napping was one of the questions the workers raised, who alleged that, despite being beneficial from a physical and mental viewpoint, as this practice is not formalized, restrictions existed with regard to its occurrence^(^ 20 ^)^.

The assessment of the scale dimensions indicated the need to reassess some items, as appropriate adjustments of the one-dimensional model were only observed when the correlation between two pairs of items was included. The questions may not have sufficient semantic distinctive capacity to capture different aspects of the need for recovery. Few studies have assessed the psychometric properties of the scale^(^ 1 ^,^ 14 ^)^ and none of the studies identified assessed the dimensional validity of the scale to permit comparisons of the presented results. Other assessments to confirm these results should be performed in other studies.

## Conclusion

The association between 2.1 to three hours of napping and the high recovery after work was confirmed in our results. Napping during the night shift increases the chance of recovery after work. In view of the inevitable nature of night shifts in hospitals, this study indicates the relevance of napping as a strategy to cope with the difficulties inherent in night work. In that sense, the main contribution of this study for nursing refers to the management of hospital work in terms of occupational health and care quality. Knowing this reality contributes to the proposal and implementation of management strategies that promote the revision of care processes and the reformulation of the human resource policy, taking into account an appropriate number of nursing professionals to avoid losses for the patients. Therefore, this research is expected to support appropriate care planning, besides helping other studies that aim to underpin more appropriate policies focused on improving the work, life and health conditions of workers, not only in nursing but nighttime workers in general.
